# CISH Expression Is Associated with Metastasis-Free Interval in Triple-Negative Breast Cancer and Refines the Prognostic Value of PDL1 Expression

**DOI:** 10.3390/cancers14143356

**Published:** 2022-07-10

**Authors:** Laurys Boudin, Alexandre De Nonneville, Pascal Finetti, Geoffrey Guittard, Jacques A. Nunes, Daniel Birnbaum, Emilie Mamessier, François Bertucci

**Affiliations:** 1 Laboratory of Predictive Oncology, Centre de Recherche en Cancérologie de Marseille, Institut Paoli-Calmettes, Aix-Marseille Université, Inserm UMR1068, CNRS UMR725, 13009 Marseille, France; laurys84@hotmail.com (L.B.); denonnevillea@ipc.unicancer.fr (A.D.N.); finettip@ipc.unicancer.fr (P.F.); daniel.birnbaum@inserm.fr (D.B.); emilie.mamessier@inserm.fr (E.M.); 2 Department of Medical Oncology, Hôpital d’Instruction des Armées Sainte-Anne, 83000 Toulon, France; 3 Department of Medical Oncology, Institut Paoli-Calmettes, 13009 Marseille, France; 4 Immunity and Cancer Team, Centre de Recherche en Cancérologie de Marseille, Institut Paoli-Calmettes, Aix-Marseille Université, Inserm UMR1068, CNRS UMR725, 13009 Marseille, France; geoffrey.guittard@inserm.fr (G.G.); jacques.nunes@inserm.fr (J.A.N.)

**Keywords:** triple-negative breast cancers, CISH, PDL1

## Abstract

**Simple Summary:**

CISH is a member of the suppressors of cytokine signaling (SOCS) family of proteins and an important negative regulator of T cells and NK cells signaling and function. In this study, analyzing 1936 triple-negative breast cancer (TNBC) clinical samples, we highlighted correlation between CISH expression and tumor features. We demonstrated that high CISH upregulation was associated with better metastasis-free interval, especially when PDL1 was also upregulated. Moreover, we showed that the two-gene model (CISH and PDL1) provided more prognostic information than each gene alone and maintained its prognostic value in multivariate analysis. Such prognostic synergy between CISH and PDL1 expressions might reinforce the therapeutic relevance of co-targeting TNBC by a combination of CISH inhibition with an immune checkpoint inhibitor blocking the PD1/PDL1 axis.

**Abstract:**

Strategies are being explored to increase the efficiency of immune checkpoint inhibitors (ICIs) targeting PD1/PDL1 in triple-negative breast cancer (TNBC), including combination with therapies inhibiting intracellular immune checkpoints such as CISH (Cytokine-induced SH2 protein). Correlation between CISH expression and TNBC features is unknown. We retrospectively analyzed *CISH* expression in 1936 clinical TNBC samples and searched for correlations with clinical variables, including metastasis-free interval (MFI). Among TNBCs, 44% were identified as “*CISH*-up” and 56% “*CISH*-down”. High expression was associated with pathological axillary lymph node involvement, more adjuvant chemotherapy, and Lehmann’s immunomodulatory and luminal AR subtypes. The “*CISH*-up” class showed longer 5-year MFI (72%) than the “*CISH*-down” class (60%; *p* = 2.8 × 10^−2^). *CISH* upregulation was associated with activation of IFNα and IFNγ pathways, antitumor cytotoxic immune response, and signatures predictive for ICI response. When *CISH* and *PDL1* were upregulated together, the 5-year MFI was 81% *versus* 52% when not upregulated (*p* = 6.21 × 10^−6^). The two-gene model provided more prognostic information than each gene alone and maintained its prognostic value in multivariate analysis. *CISH* expression is associated with longer MFI in TNBC and refines the prognostic value of *PDL1* expression. Such observation might reinforce the therapeutic relevance of combining CISH inhibition with an anti-PD1/PDL1 ICI.

## 1. Introduction

In contrast with other solid tumors, such as melanoma or lung cancer, the role of immunity in breast cancer has emerged only recently. First, the favorable prognostic impact of several immune features in the tumor was demonstrated: tumor-infiltrating lymphocytes (TILs) [1,2], immune expression signatures, notably for ER-negative, highly proliferative tumors [3,4,5,6], or expression of PDL1 in triple-negative breast cancer (TNBC) [7]. However, these prognostic features remain imperfect in front of the known heterogeneity of TNBC. Second, randomized clinical trials evaluating immune checkpoint inhibitors (ICIs) in combination with chemotherapy in patients with TNBC reported positive results [8] and led to the FDA-approvals of pembrolizumab (anti-PD1) and atezolizumab (anti-PDL1) in advanced disease [9,10] and pembrolizumab in early disease [11]. However, not all patients derive benefit from these ICIs dedicated to improving antitumor cytotoxic immune responses, and many patients develop resistance to these treatments. These limitations emphasize the need to identify new cells and molecules that could be exploited in the next generation of immunotherapy [12].

Different strategies are being explored to increase the efficiency of ICIs [8]. One of them is the combination with therapies inhibiting intracellular immune checkpoints, such as CISH (Cytokine-induced SH2 protein). CISH is one of the eight members of the suppressors of cytokine signaling (SOCS) family of proteins [13] and an important negative regulator of T cells and NK cells signaling and function. The inhibition of CISH in mouse anti-tumor CD8 T-lymphocytes resulted in a marked increase in the ability of these lymphocytes to mediate tumor regression in vivo [14,15,16]. These data led to the launching of an ongoing phase I/II clinical trial (NCT04426669) dedicated to patients with gastro-intestinal cancer, in which *CISH* is inactivated using CRISPR gene editing in neoantigen-specific TIL. In vivo, the *CISH* KO in T-cells increased PD1 expression and the adoptive transfer of *Cish*-KO T-cells synergistically combined with PD1 antibody blockade resulting in durable tumor regression and survival in a preclinical animal model [17]. CISH is also a potent checkpoint in NK cell-mediated tumor immunity [18]. CISH deletion in NK cells increases ex-vivo proliferation, functions and signaling activation of several signaling pathways such as cytokines and Natural cytotoxicity receptors (NCR). In vivo CISH absence favors NK cell numbers to the tumor burden, optimizes their killing properties and limits NK cell exhaustion leading to primary breast cancer growth in addition to superior control of spontaneous tumor metastasis [19]. These data suggested possibilities for immunotherapies directed at blocking CISH function and their potential for improving the efficacy of ICIs in combination. This was confirmed in vivo in combination with anti-PDL1 and anti-CTLA4, as well with IL-2 and type I interferon in term of protection from tumor initiation and metastasis [18,20]. Thus, CISH inhibition might improve the efficiency of current ICIs.

To our knowledge, only one study [21] in the literature analyzed *CISH* expression in mammary clinical samples. In this small series (17 breast cancer samples and 3 normal breast samples), no correlation was searched with tumor features. In order to fill this gap, and given the potential therapeutic relevance of CISH, we analyzed *CISH* expression in 1936 TNBC clinical samples. We searched for correlations between *CISH* expression and molecular and clinicopathological data, including metastasis-free interval. We also investigated whether *CISH* expression could refine the known prognostic value of *PDL1* expression in TNBC.

## 2. Materials and Methods

### 2.1. Breast Cancer Samples

Our own data set of clinical samples included 353 cases representing pre-treatment invasive carcinomas from patients with non-metastatic and non-inflammatory disease at diagnosis [22]. The study was approved by our institutional review board (the Institut Paoli-Calmettes (IPC) “Comité d’Orientation Stratégique”, n°2010-012) and each patient had given a written informed consent for research use. We pooled this with 35 publicly available data sets comprising at least one probe set representing *CISH*. These sets were collected from the National Center for Biotechnology Information (NCBI)/Genbank GEO and ArrayExpress databases, and authors’ website (Supplementary Table S1) [23]. The final pooled data set included 8982 non-redundant, non-metastatic, non-inflammatory, primary, invasive breast cancers and 501 normal breast samples with *CISH* mRNA expression and clinicopathological data available.

### 2.2. Gene Expression Data Analysis

Our own gene expression data set (clinical normal and cancer samples) had been generated using Affymetrix U133 Plus 2.0 human microarrays (Affymetrix^®^, Santa Clara, CA, USA) as previously described [24]. All data were MIAME compliant and deposited in the GEO databases (GSE31448). The other pooled data sets had been profiled using DNA microarrays or RNA-sequencing (Supplementary Table S1). The pre-analytic processing of gene expression data required several successive steps and was done as previously described [25]. CISH expression in tumors (T) was measured as discrete value after comparison with median expression in normal breast samples (NB): upregulation, thereafter designated “CISH-up” was defined by a T/NB ratio >1 and no upregulation (“CISH-down”) by a T/NB ratio <1. To avoid biases related to immunohistochemistry (IHC) analyses across different institutions, estrogen receptor (ER), progesterone receptor (PR) and ERBB2 expression status were defined at the mRNA level using gene expression data of their respective gene, *ESR1*, *PGR* and *ERBB2* using a 2-component Gaussian mixture distribution model. A total of 1936 clinical cancer samples were defined as TNBC and were included in the present study. The six Lehmann’s subtypes were defined as described [26]. Given the involvement of CISH in immunity, we applied, in each data set separately, several multigene immune classifiers, including the metagenes associated with the T-cell-inflamed signature (TIS) [27], the tertiary lymphoid structures (TLS) signature [28], the signatures of 24 different innate and adaptative immune cell subpopulations defined by Bindea et al. [29], the cytolytic activity score [30], the pathway activation score of IFNα, IFNγ, STAT3, TGFβ and TNFα [31], and the Immune Constant of Rejection (ICR) signature [6].

### 2.3. Statistical Analysis

Correlations between the tumor classes and clinicopathological and molecular features were analyzed using the t-test or the Fisher’s exact test (variables with 2 groups) when appropriate. Metastasis-free interval (MFI) was calculated from the date of diagnosis until the date of distant relapse. Follow-up was measured from the date of diagnosis to the date of last news for event-free patients. Uni- and multivariate analyses for MFI were done using Cox regression analysis (Wald test). Variables tested in univariate analyses included patients’ age at time of diagnosis (≤50 years vs >50), pathological tumor size (pT: pT1 vs. pT2 vs. pT3), pathological axillary lymph node status (pN: negative vs. positive), pathological grade (1 vs. 2 vs. 3), pathological type (ductal vs. lobular vs. other), Lehmann’s subtypes (basal-like 1 vs. basal-like 2 vs. mesenchymal vs. mesenchymal stem-like vs. immunomodulatory vs. luminal androgen receptor) and CISH expression-based class (“up” vs. “down”). Variables with a *p*-value < 0.05 in univariate analysis were tested in multivariate analysis. The likelihood ratio (LR) tests were used to assess the prognostic information provided beyond that of each gene (*CISH* and *PDL1*), assuming an X2 distribution. Changes in the LR values (LR-ΔX2) quantified the relative amount of information of one model compared with another. Statistical analysis was done using the survival package (version 2.43) in the R software (version 3.5.2). We followed the reporting REcommendations for tumor MARKer prognostic studies (REMARK criteria). A diagram of analytic workflow (Supplementary Figure S1) summarizes all analyses.

## 3. Results

### 3.1. CISH Expression in TNBC and Clinicopathological Features

*CISH* expression was analyzed in 501 normal mammary tissue samples and 1936 primary TNBC clinical samples. Expression was heterogeneous through cancer samples (Figure S1), and as compared to normal breast expression, 44% of TNBCs were defined as “*CISH*-up” and 56% as “*CISH*-down”. Such heterogeneity allowed to search for correlations with clinicopathological features (Table 1). Overall, “*CISH*-up” tumors were associated (Fisher’s exact test) with pathological axillary lymph node involvement (*p* = 6.33 × 10^−3^) and more delivery of adjuvant chemotherapy (*p* = 7.22 × 10^−4^). No correlation was found with patients’ age, pathological tumor type, size and grade. Finally, “*CISH*-up” TNBC samples were more frequently immunomodulatory (IM) and luminal AR (LAR) subtype, whereas “*CISH*-down” tumors were more frequently basal-like1 (BL1) and mesenchymal (M) subtype (*p* = 6.32 × 10^−39^).

### 3.2. CISH Expression and Metastasis-Free Interval in TNBC

**Figure 1:**
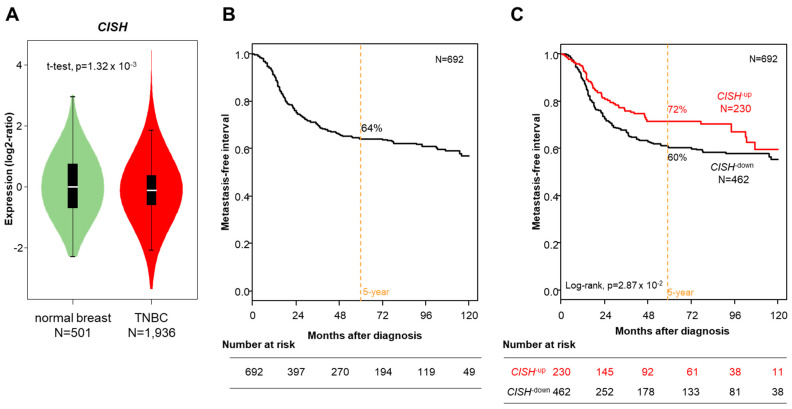
CISH expression in TNBC and metastasis-free interval. (**A**).Violin plots of CISH mRNA expression in normal breast samples and TNBC samples. The *p*-value is for the Student t-test. (**B**) Kaplan–Meier MFI curve in the 692 informative patients with TNBC. (**C**) Similar to B, but according to the CISH expression-based class (“CISH-up” and “CISH-down”).

We assessed the prognostic value of *CISH* expression in the 692 TNBC patients with available MFI data. With a median follow up of 39 months (range, 1–286), 207 events (30%) occurred, and the 5-year MFI was 64% (95% CI, 60–68; Figure 1A). As shown in Figure 1B and Table 1, the 5-year MFI was different among the two CISH classes: 72% (95% CI, 65–79) in the “*CISH*-up” class *versus* 60% (95% CI, 55–66) in the “*CISH*-down” class (*p* = 2.87 × 10^−2^, log-rank test). In univariate analysis (Table 2), the delivery of adjuvant chemotherapy (*p* = 4.03 × 10^−2^), the Lehmann’s molecular subtypes (*p* = 4.75 × 10^−3^), and the “*CISH*-up” class (*p* = 2.92 × 10^−2^) were associated with longer MFI. The hazard ratio (HR) for metastatic relapse was 0.71 (95% CI, 0.53–0.97) in the “*CISH*-up” class as compared with the “*CISH*-down” class (Wald test). However, in multivariate analysis, the CISH class did not remain significant (*p* = 0.909), by contrast to chemotherapy (*p* = 4.80 × 10^−2^) and Lehmann’s immune (*p* = 1.01 × 10^−3^) and basal-like 1 (*p* = 1.04 × 10^−2^) subtypes that were associated with lesser risk of event when compared with the mesenchymal subtype. The same result was observed in uni- and multivariate analyses when considering CISH expression as continuous variable or as discrete variable using a cut-off equal to the median expression level in TNBC samples (Supplementary Table S2).

### 3.3. CISH Expression and Immune Features in TNBC

**Figure 2:**
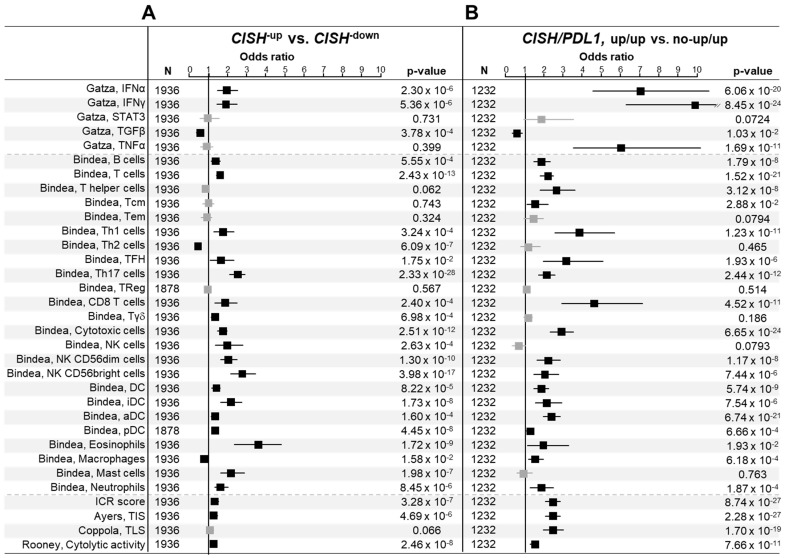
Association of CISH expression-based TNBC classes with immune variables. Forest plots showing the Odds Ratios (log10) of 5 Gatza’s activation pathways, 24 Bindea’s immune cell types signatures and 4 immune signatures in the “CISH-up” vs. “CISH-down” classes comparison (**A**) and in the “CISH-up/PDL1-up” vs. “no-CISH-up/PDL1-up” groups (**B**). The black squares correspond to significant variables and the grey ones to non-significant variables.

Next, we investigated whether *CISH* expression was associated with immunity-related parameters in TNBC samples (Figure 2A). We found higher probability of activation of anti-tumor IFNα and IFNγ pathways and lower probability of activation of the pro-tumor TGFβ pathway in the “*CISH*-up” class, as compared to the “*CISH*-down” class. In total, 18 of the 24 Bindea’s signatures for immune cell subsets were significantly enriched in the “*CISH*-up” class, including signatures for adaptative immune cells (B cells, T cells, Th1 cells, cytotoxic T cells, and CD8+ T cells, TFH cells, and Th17 cells) and innate immune cells (Tγδ, NK cells, NK^CD56dim^ cells, NK^CD56bright^ cells, eosinophils, mast cells, and neutrophils). Cell subsets involved in antigen presentation, such as dendritic cells (DC), aDC (activated DC), iDC (immature DC), pDC (plasmacytoid DC), and B cells, were higher in the “*CISH*-up” class. The “*CISH*-up” class displayed higher ICR score and Rooney’s cytolytic activity score, reflect of an antitumor cytotoxic immune response, than the “CISH-down” class. Two signatures predictive for response to ICI, the T cell-inflamed signature (TIS) and the tertiary lymphoid structure (TLS) score, were also higher in the “*CISH*-up” class. Among the T-helper cells, the Th1/Th2 ratio was higher in the “*CISH*-up” class. However, the most differential Th subset between the “*CISH*-up” and “*CISH*-down” classes was the Th17 subset, which showed a “*CISH*-up” class/“*CISH*-down” class odds ratio (OR) higher than that of the Th1 cells. Among the innate immune cells, the more discriminant subset was the eosinophils, also known to exert, as for the Th17 subset, conflicting functions according to the tumor microenvironment.

### 3.4. Prognostic Synergy of PDL1 and CISH Expression in TNBC

**Figure 3:**
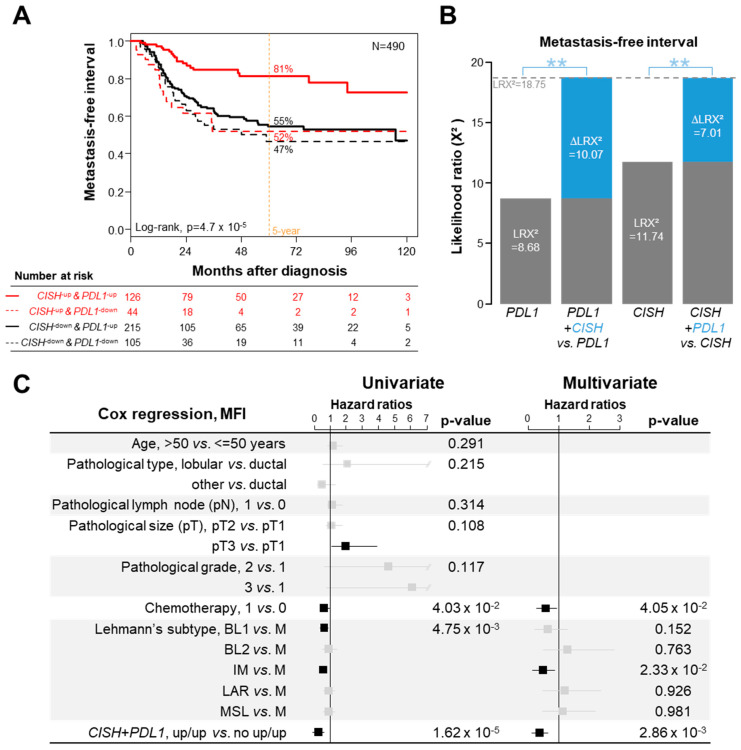
Prognostic synergy of CISH and PDL1 expression in TNBC. (**A**) Kaplan–Meier MFI curves in the 490 informative TNBC patients with documented CISH and PDL1 expression, according to four classes defined by the expression status of both CISH and PDL1 (color scale to the left of table). (**B**) Comparison of the prognostic information for MFI of several models based on unique and combined expression status of CISH and PDL1. The values are given for prognostic information of each variable colored in grey (PDL1 and CISH) on its own (LR-χ2) and when added to the other variable colored in blue (ΔLR-χ2). The blue stars indicate the significance of ΔLR- χ2 (** indicates *p* ≤ 0.01). (**C**) Uni- and multivariate Cox analyses for MFI, including the CISH/PDL1-based classification.

Given the known independent prognostic value of PDL1 expression in TNBC and our prognostic results with *CISH*, we tested the hypothesis of a predictive complementarity of both markers. *CISH* and *PDL1* mRNA expressions were available in 1232 samples, including 490 informative for MFI. As shown in Figure 3A, the patients with upregulation of both genes (“*CISH*-up/*PDL1*-up” group) displayed 81% 5-year MFI (95 Cl, 74–90), whereas patients with downregulation of both genes displayed 47% 5-year MFI (95 Cl, 34–63) and those with opposite deregulation of both genes (up/down, and down/up) displayed respective 52% (95 CI, 37–74) and 55% (95 CI, 47–64) 5-year MFI (*p* = 4.7 × 10^−5^, log-rank test). Since these last three patients’ groups showed similar MFI, they were merged and thereafter designated “no-CISH-up/PDL1-up” group. The 5-year MFI of this latter was 52% (95 Cl, 46–59) *versus* 81% (95 Cl, 74–90) in the “CISH-up/PDL1-up” group (*p* = 6.21 × 10^−6^, log-rank test). The prognostic synergy of expression of both genes was confirmed using the likelihood ratio (LR) test: the two-gene combination model provided more prognostic information than *PDL1* alone (ΔLR-X2 = 10.06, *p* = 1.51 × 10^−3^) and than *CISH* alone (ΔLR-X2 = 7.01, *p* = 8.12 × 10^−3^; Figure 3B). In univariate analysis (Figure 3C), the HR for metastatic relapse in the “*CISH*-up/*PDL1*-up” group as compared with the “no-*CISH*-up/*PDL1*-up” group was 0.34 (95% CI, 0.21–0.56; *p* = 1.62 × 10^−5^, Wald test). In multivariate analysis, the *CISH*/*PDL1* status remained significant (*p* = 2.86 × 10^−3^, Wald test), as did the Lehmann’s immune subtype, suggesting independent prognostic value. In order to estimate the robustness of these two prognostic groups, we randomly split the 490 informative samples in two sets, training (244 samples) and validation (246 samples) sets (Supplementary Figure S2). In the training set, the model showed a significant difference between the 5-year MFI of the “*CISH*-up/*PDL1*-up” group (81%, 95 CI 70–94) and the “no-*CISH*-up/*PDL1*-up” group (58%, 95 CI 50–69; *p* = 5.25 × 10^−3^, log-rank test). Importantly, this grouping maintained its prognostic value in the validation set, with 82% 5-year MFI in the “*CISH*-up/*PDL1*-up” group (95 CI 72–94) *versus* 46% in the “no-*CISH*-up/*PDL1*-up” group (95 CI 38–56; *p* = 7.51 × 10^−4^, log-rank test).

The Figure 2B shows the correlations between the two tumor groups and the immunity-related parameters: most of parameters were positively associated with the “*CISH-up*/*PDL1-up*” group, with OR superior to those observed in Figure 2A. Notably, the more discriminant odds ratio (OR) of the “*CISH*-up/*PDL1*-up” group was now obtained for IFNα, IFNγ and TNFα pathways, the three major cytokines responsible for cytotoxic function, the Th1 cell and the CD8 cell subsets. This was confirmed at the functional level with the significant enrichment for ICR, TIS, TLS and cytolytic scores. Altogether, these data suggested a permissive function of PDL1 and CISH in the metastatic process of TNBC.

## 4. Discussion

The co-targeting of cell surface immune checkpoint proteins (PD1 and PDL1) along with intracellular checkpoint molecules such as CISH is a promising approach [12], and might improve the results of ICIs in TNBC. Our objective was to document *CISH* expression in a large series of TNBC clinical samples and to search for correlations with tumor features and an eventual synergy with *PDL1* expression. We show that high *CISH* expression is associated with better MFI in TNBC, and refines the prognostic value of *PDL1* expression.

During the last few years, very few results have been published regarding expression of SOCS proteins, including CISH, in human cancers [32]. In breast cancer, only one study analyzed CISH expression in only 17 cases [21]. Our analysis at the mRNA level allowed us to not only work on a large series of TNBC samples, but also to search for associations with expression of immune signatures while avoiding the classical limitations of immunohistochemistry (standardization, positivity cut-off, interpretation subjectivity). We found heterogeneous expression of *CISH* in TNBC samples, as reported at the protein and mRNA levels in the unique and small series of 17 breast cancers and 3 normal breast tissues [21]. Overall, in the whole population (1936 TNBC samples *versus* 501 normal breast samples), *CISH* expression was lower in the TNBC samples than in the normal samples. As discrete value and using a non-stringent cut-off (expression superior or inferior to the median expression in NB samples), 44% of TNBCs were respectively defined as “*CISH*-up” and 56% as “*CISH*-down”. “*CISH-up*” tumors were associated with pathological lymph node involvement, without correlation with patients’ age, pathological tumor type, size and grade. Except the small above-quoted series, no data about *CISH* expression in breast cancer are available in the literature. By contrast, data are available regarding other SOCS family genes [33,34,35]. Sasi et al. did not find difference in expression for SOCS1 to 7 in 127 breast cancer samples and 31 normal breast samples [33] and higher expression of SOCS1, 3, 4 and 7 was associated with earlier tumor stage and better clinical outcome. Ghafouri-Fard et al. reported downregulation of SOCS1–3 and SOCS5 genes in tumor tissues (N = 44) compared with the corresponding adjacent non-cancerous tissues, and *SOCS1* and *SOCS2* genes were associated with higher tumor grade [35]. In an analysis of 1109 breast cancer samples and 113 normal breast samples [34], SOCS2 and SOCS3 showed lower expression levels in cancer tissues than in normal tissues, and patients with high expression of SOCS2, SOCS3 and SOCS4 exhibited better outcome. For the first time, we showed that *CISH* was differentially expressed across the six TNBC molecular subtypes, with higher expression in the IM and LAR subtypes and lower expression in the BL1 and M subtypes.

A total of 692 TNBC patients were informative for MFI, and none of them had been treated by (neo)adjuvant immunotherapy. *CISH* upregulation was associated with longer MFI in univariate analysis. Strikingly, the two MFI curves, “*CISH*-up” and “*CISH*-down”, which gradually diverged with time, converged from 96 months of follow-up leading to similar 10-year MFI. This suggested a suddenly higher rate of very late relapses in the “*CISH*-up” group than in the “*CISH*-down” group. That was not related to the pathological grade that was well balanced between the two groups; currently, we do not have any explanation for this observation that will deserve confirmation in larger series. To date, no data are available regarding the prognostic value of *CISH* in breast cancer, whereas the expression of certain SOCS genes has already been associated with better prognosis [33,34]. Such favorable prognostic value of CISH expression seems paradoxical given its known immunosuppressive role, but this paradox has already been reported in TNBC for other immunosuppressive molecules such as PDL1 [7] or IDO [36], Meaning it likely represents a negative feedback mechanism following effective antitumor response. Indeed, we observed an enrichment of the “*CISH*-up” tumors in several signatures suggestive of a higher antitumor T cell response as compared with the “*CISH*-down” tumors. However, the higher relative abundance of Th17 cells in the “*CISH*-up” *versus* “*CISH*-down” class, as compared with that of Th1 cells, suggested that this response was not optimal in the “*CISH*-up” class. Such limitation might contribute to the loss of prognostic value of *CISH* in the multivariate analysis when confronted to the Lehmann’s molecular subtypes that include the immunomodulatory subtype. Indeed, Th17 are highly plastic cells capable of re-differentiation [37], and their effect in cancer is ambiguous and dependent on the chosen re-differentiation path: either as pro-tumor Treg or Th2-like cells or anti-tumor Th1 cells [38]. It is likely that the density and proportion of cytokines and chemokines existing in the tumor environment, and the presence and impact of other tumor-infiltrating immune cells determine the orientation of Th17 final polarization. A combination with ICI might thus help re-polarize these “undetermined” Th17 cells into efficient Th1 cells. Interestingly and corroborating this hypothesis, we showed the prognostic synergy of CISH expression with that of PDL1 and higher association with stronger antitumor immune function. As expected, the “*PDL1*-up” class was associated with better survival than the “*PDL1*-down” class in our TNBC series. But, each marker, *CISH* and *PDL1*, improved the prognostic value of the other one, and the *CISH* + *PDL1* combination predicted MFI better than did each immune marker alone. It resulted in a large MFI difference between the two groups with 52% 5-year MFI in the “no-*CISH*-up/*PDL1*-up” group *versus* 81% in the “*CISH*-up/*PDL1*-up” group. In multivariate analysis, this two-gene combination was independent from the Lehmann’s subtypes. In this case, the antitumor immune response in the “*CISH*-up/*PDL1*-up” group was likely much more optimal than it was in the “*CISH*-up” class, as demonstrated by the much stronger “*CISH*-up” class/“*CISH*-down” class odds ratio (OR) for the Th1 cells than for the Th17 cells. From a therapeutic point of view, because this immune response is partially inhibited by both immune checkpoints (CISH and PDL1), their therapeutic blockade should allow the reactivation of inhibited T-cells to increase the antitumor immune response, and improve the clinical outcome observed in responder patients. Recent clinical trials such as Keynote-522 have clearly showed the strong clinical benefit of adding pembrolizumab to neo-adjuvant chemotherapy, notably in PDL1-positive patients [11,39]. Thus, the CISH-up/PDL1-up patients, who displayed an 81% 5-year MFI that deserves to be improved, might benefit from a dual blockade of CISH and PDL1. Indeed, several studies highlighted the inhibition of CISH as a method of unleashing the NK cell [18,19,40,41] and T cell [15,16,17] antitumor response, leading to increase of IFNγ production, cytotoxicity, and PDL1 expression. These in vivo data highlighted the potential of inhibiting the CISH intracellular immune checkpoint in combination with ICIs to overcome ICI resistance. Delconte et al. demonstrated that combining anti-PD1 and anti-CTLA4 ICIs with CISH^-/-^ NK-cells drastically reduced lung metastasis in vivo as compared to the IgG control and CISH^+/+^ NK-cells alone in the adoptive transfer model, highlighting the potential benefit of such combination [18]. This benefit was further demonstrated by the same team using pre-clinical models of sarcoma, melanoma and prostate carcinoma [20]. Phase-I clinical trials targeting CISH and PDL1 are ongoing and planned in solid tumors [14,42]. The prognostic complementarity between *CISH* and *PDL1* that we report here reinforces the relevance of their co-inhibition.

## 5. Conclusions

We showed that *CISH* expression is associated with longer MFI in patients with TNBC and refines the prognostic value of *PDL1* expression. To our knowledge, with 1936 cases analyzed, this series is the largest one reported in breast cancer. The main strength of our study lies in the number of samples analyzed, but also their homogeneity (only TN subtype), and analysis of correlations with many immune variables. Limitations include its retrospective nature and associated biases, and the absence of analysis at the protein level. If confirmed at the protein level, such prognostic synergy between *CISH* and *PDL1* expressions would reinforce the therapeutic relevance of co targeting TNBC by a combination of CISH inhibition with an ICI blocking the PD1/PDL1 axis. Further pre-clinical data and the launching of clinical trials testing this hypothesis are warranted in TNBC. Besides this prognostic role, another crucial objective will be to assess CISH expression as an eventual predictive biomarker that could help to guide ICI therapy in TNBC.

## Figures and Tables

**Table 1:** Clinico-pathological characteristics.

	N	All	CISH Class		
			Down	Up	*p*-Value
Age at diagnosis (years)					0.480
≤50	726	726 (47%)	570 (46%)	156 (49%)	
>50	824	824 (53%)	660 (54%)	164 (51%)	
Pathological type					0.359
ductal	814	814 (83%)	683 (84%)	131 (81%)	
lobular	31	31 (3%)	23 (3%)	8 (5%)	
other	133	133 (14%)	111 (14%)	22 (14%)	
Pathological lymph node (pN)					6.33 × 10^−3^
negative	662	662 (57%)	576 (59%)	86 (48%)	
positive	499	499 (43%)	404 (41%)	95 (52%)	
Pathological size (pT)					0.543
pT1	344	344 (32%)	294 (32%)	50 (31%)	
pT2	606	606 (56%)	519 (56%)	87 (54%)	
pT3	139	139 (13%)	114 (12%)	25 (15%)	
Pathological grade					0.482
1	36	36 (3%)	26 (2%)	10 (3%)	
2	243	243 (17%)	184 (17%)	59 (19%)	
3	1114	1114 (80%)	873 (81%)	241 (78%)	
Lehmann’s subtypes *					6.32 × 10^−39^
BL1	308	308 (16%)	220 (20%)	88 (10%)	
BL2	144	144 (7%)	80 (7%)	64 (8%)	
IM	395	395 (20%)	168 (15%)	227 (27%)	
LAR	314	314 (16%)	110 (10%)	204 (24%)	
M	447	447 (23%)	339 (31%)	108 (13%)	
MSL	328	328 (17%)	169 (16%)	159 (19%)	
Chemotherapy delivery					
no	364	364 (28%)	317 (30%)	47 (19%)	7.22 × 10^−4^
yes	920	920 (72%)	724 (70%)	196 (81%)	
Follow-up median, months (min-max)	692	39 (1–286)	36 (1–286)	43 (1–181)	0.076
MFI event, N (%)	692	207 (30%)	149 (32%)	58 (25%)	0.064
5-year MFI (95%CI)	692	64% (60–68)	60% (55–66)	72% (65–79)	2.87 × 10^−2^

* Lehmann’s TNBC subtypes, BL1—Basal-like 1; BL2—Basal-like 2; IM—Immunomodulatory; LAR—Luminal Androgen Receptor; M—Mesenchymal; MSL—Mesenchymal Stem-like.

**Table 2:** Uni- and multivariate analyses for MFI.

MFI, TNBC		Univariate			Multivariate		
		N	HR [95% CI]	*p*-Value	N	HR [95% CI]	*p*-Value
Age at diag. (years)	>50 vs. ≤50	534	1.22 [0.84–1.78]	0.291			
Pathological type	lobular vs. ductal	343	2.13 [0.52–8.84]	0.215			
	other vs. ductal		0.53 [0.21–1.34]				
Pathological lymph node (pN)	positive vs. negative	524	1.21 [0.83–1.77]	0.314			
Pathological size (pT)	pT2 vs. pT1	475	1.13 [0.72–1.76]	0.108			
	pT3 vs. pT1		1.99 [1.03–3.83]				
Pathological grade	2 vs. 1	343	4.69 [0.63–34.95]	0.117			
	3 vs. 1		6.16 [0.86–44.31]				
Chemotherapy delivery	yes vs. no	497	0.67 [0.46–0.98]	4.03 × 10^−2^	497	0.68 [0.46–1.00]	4.80 × 10^−2^
Lehmann’s subtypes *	BL1 vs. M	692	0.59 [0.38–0.90]	4.75 × 10^−3^	497	0.47 [0.26–0.84]	1.04 × 10^−2^
	BL2 vs. M		0.87 [0.52–1.45]		497	0.65 [0.31–1.35]	0.249
	IM vs. M		0.44 [0.29–0.68]		497	0.37 [0.20–0.67]	1.01 × 10^−3^
	LAR vs. M		0.86 [0.57–1.30]		497	0.60 [0.32–1.12]	0.107
	MSL vs. M		0.74 [0.47–1.18]		497	0.72 [0.40–1.30]	0.278
CISH class	up vs. down	692	0.71 [0.53–0.97]	2.92 × 10^−2^	497	0.98 [0.63–1.51]	0.909

* Lehmann’s TNBC subtypes, BL1—Basal-like 1; BL2—Basal-like 2; IM—Immunomodulatory; LAR—Luminal Androgen Receptor; M—Mesenchymal; MSL—Mesenchymal Stem-like.

## Data Availability

All data sets are publicly available and references are described in Table S1.

## Supplementary Materials

The following supporting information can be downloaded at: https://www.mdpi.com/article/10.3390/cancers14143356/s1, Supplementary Table S1: List of breast cancer data sets included in the study. Supplementary Figure S1: Diagram of analytic workflow. Supplementary Table S2: Uni- and multivariate analyses for MFI using CISH expression as continuous variable or as discrete variable with a cut-off equal to the median expression level in TNBC samples. Supplementary Figure S2: Robustness of the prognostic synergy of CISH and PDL1. The 490 informative samples were divided into two sets, training (244 samples) and validation (246 samples) sets, in which the prognostic value of the CISH/PDL1 expression-based model was tested independently.

## References

[1] Denkert C., Loibl S., Noske A., Roller M., Müller B.M., Komor M., Budczies J., Darb-Esfahani S., Kronenwett R., Hanusch C. (2010). Tumor-Associated Lymphocytes as an Independent Predictor of Response to Neoadjuvant Chemotherapy in Breast Cancer. J. Clin. Oncol..

[2] Ali H.R., Provenzano E., Dawson S.-J., Blows F.M., Liu B., Shah M., Earl H.M., Poole C.J., Hiller L., Dunn J.A. (2014). Association between CD8+ T-Cell Infiltration and Breast Cancer Survival in 12,439 Patients. Ann. Oncol..

[3] Rody A., Holtrich U., Pusztai L., Liedtke C., Gaetje R., Ruckhaeberle E., Solbach C., Hanker L., Ahr A., Metzler D. (2009). T-Cell Metagene Predicts a Favorable Prognosis in Estrogen Receptor-Negative and HER2-Positive Breast Cancers. Breast Cancer Res..

[4] Teschendorff A.E., Miremadi A., Pinder S.E., Ellis I.O., Caldas C. (2007). An Immune Response Gene Expression Module Identifies a Good Prognosis Subtype in Estrogen Receptor Negative Breast Cancer. Genome Biol..

[5] Sabatier R., Finetti P., Mamessier E., Raynaud S., Cervera N., Lambaudie E., Jacquemier J., Viens P., Birnbaum D., Bertucci F. (2011). Kinome Expression Profiling and Prognosis of Basal Breast Cancers. Mol. Cancer.

[6] Bertucci F., Finetti P., Simeone I., Hendrickx W., Wang E., Marincola F.M., Viens P., Mamessier E., Ceccarelli M., Birnbaum D. (2018). The Immunologic Constant of Rejection Classification Refines the Prognostic Value of Conventional Prognostic Signatures in Breast Cancer. Br. J. Cancer.

[7] Sabatier R., Finetti P., Mamessier E., Adelaide J., Chaffanet M., Ali H.R., Viens P., Caldas C., Birnbaum D., Bertucci F. (2015). Prognostic and Predictive Value of PDL1 Expression in Breast Cancer. Oncotarget.

[8] Emens L.A., Adams S., Cimino-Mathews A., Disis M.L., Gatti-Mays M.E., Ho A.Y., Kalinsky K., McArthur H.L., Mittendorf E.A., Nanda R. (2021). Society for Immunotherapy of Cancer (SITC) Clinical Practice Guideline on Immunotherapy for the Treatment of Breast Cancer. J. Immunother. Cancer.

[9] Cortes J., Cescon D.W., Rugo H.S., Nowecki Z., Im S.-A., Yusof M.M., Gallardo C., Lipatov O., Barrios C.H., Holgado E. (2020). Pembrolizumab plus Chemotherapy versus Placebo plus Chemotherapy for Previously Untreated Locally Recurrent Inoperable or Metastatic Triple-Negative Breast Cancer (KEYNOTE-355): A Randomised, Placebo-Controlled, Double-Blind, Phase 3 Clinical Trial. Lancet.

[10] Emens L.A., Adams S., Barrios C.H., Diéras V., Iwata H., Loi S., Rugo H.S., Schneeweiss A., Winer E.P., Patel S. (2021). First-Line Atezolizumab plus Nab-Paclitaxel for Unresectable, Locally Advanced, or Metastatic Triple-Negative Breast Cancer: IMpassion130 Final Overall Survival Analysis. Ann. Oncol..

[11] Schmid P., Cortes J., Pusztai L., McArthur H., Kümmel S., Bergh J., Denkert C., Park Y.H., Hui R., Harbeck N. (2020). Pembrolizumab for Early Triple-Negative Breast Cancer. N. Engl. J. Med..

[12] Kumar S., Sarthi P., Mani I., Ashraf M.U., Kang M.-H., Kumar V., Bae Y.-S. (2021). Epitranscriptomic Approach: To Improve the Efficacy of ICB Therapy by Co-Targeting Intracellular Checkpoint CISH. Cells.

[13] Yoshimura A., Ito M., Chikuma S., Akanuma T., Nakatsukasa H. (2018). Negative Regulation of Cytokine Signaling in Immunity. Cold Spring Harb. Perspect. Biol..

[14] Palmer D., Webber B., Patel Y., Johnson M., Kariya C., Lahr W., Parkhurst M., Gartner J., Prickett T., Lowery F. (2020). 333 Targeting the Apical Intracellular Checkpoint CISH Unleashes T Cell Neoantigen Reactivity and Effector Program. J. Immunother. Cancer.

[15] Guittard G., Dios-Esponera A., Palmer D.C., Akpan I., Barr V.A., Manna A., Restifo N.P., Samelson L.E. (2018). The Cish SH2 Domain Is Essential for PLC-γ1 Regulation in TCR Stimulated CD8+ T Cells. Sci. Rep..

[16] Palmer D.C., Guittard G.C., Franco Z., Crompton J.G., Eil R.L., Patel S.J., Ji Y., Van Panhuys N., Klebanoff C.A., Sukumar M. (2015). Cish Actively Silences TCR Signaling in CD8+ T Cells to Maintain Tumor Tolerance. J. Exp. Med..

[17] Palmer D.C., Webber B.R., Patel Y., Johnson M.J., Kariya C.M., Lahr W.S., Parkhurst M.R., Gartner J.J., Prickett T.D., Lowery F.J. (2020). Internal Checkpoint Regulates T Cell Neoantigen Reactivity and Susceptibility to PD1 Blockade. bioRxiv.

[18] Delconte R.B., Kolesnik T.B., Dagley L.F., Rautela J., Shi W., Putz E.M., Stannard K., Zhang J.-G., Teh C., Firth M. (2016). CIS is a Potent Checkpoint in NK Cell-Mediated Tumor Immunity. Nat. Immunol..

[19] Bernard P.-L., Delconte R.B., Pastor S., Laletin V., Silva C.C.D., Goubard A., Josselin E., Castellano R., Krug A., Vernerey J. (2021). Targeting CISH Enhances Natural Cytotoxicity Receptor Signaling and Reduces NK Cell Exhaustion to Improve Solid Tumor Immunity. bioRxiv.

[20] Putz E.M., Guillerey C., Kos K., Stannard K., Miles K., Delconte R.B., Takeda K., Nicholson S.E., Huntington N.D., Smyth M.J. (2017). Targeting Cytokine Signaling Checkpoint CIS Activates NK Cells to Protect from Tumor Initiation and Metastasis. Oncoimmunology.

[21] Raccurt M., Tam S.P., Lau P., Mertani H.C., Lambert A., Garcia-Caballero T., Li H., Brown R.J., McGuckin M.A., Morel G. (2003). Suppressor of Cytokine Signalling Gene Expression Is Elevated in Breast Carcinoma. Br. J. Cancer.

[22] Sabatier R., Finetti P., Adelaide J., Guille A., Borg J.-P., Chaffanet M., Lane L., Birnbaum D., Bertucci F. (2011). Down-Regulation of ECRG4, a Candidate Tumor Suppressor Gene, in Human Breast Cancer. PLoS ONE.

[23] de Nonneville A., Finetti P., Mamessier E., Bertucci F. (2022). RE: NDRG1 in Aggressive Breast Cancer Progression and Brain Metastasis. J. Natl. Cancer Inst..

[24] Bertucci F., Finetti P., Cervera N., Charafe-Jauffret E., Buttarelli M., Jacquemier J., Chaffanet M., Maraninchi D., Viens P., Birnbaum D. (2009). How Different Are Luminal A and Basal Breast Cancers?. Int. J. Cancer.

[25] Gonçalves A., Finetti P., Sabatier R., Gilabert M., Adelaide J., Borg J.-P., Chaffanet M., Viens P., Birnbaum D., Bertucci F. (2011). Poly(ADP-Ribose) Polymerase-1 mRNA Expression in Human Breast Cancer: A Meta-Analysis. Breast Cancer Res. Treat..

[26] Lehmann B.D., Bauer J.A., Chen X., Sanders M.E., Chakravarthy A.B., Shyr Y., Pietenpol J.A. (2011). Identification of Human Triple-Negative Breast Cancer Subtypes and Preclinical Models for Selection of Targeted Therapies. J. Clin. Investig..

[27] Ayers M., Lunceford J., Nebozhyn M., Murphy E., Loboda A., Kaufman D.R., Albright A., Cheng J.D., Kang S.P., Shankaran V. (2017). IFN-γ-Related mRNA Profile Predicts Clinical Response to PD-1 Blockade. J. Clin. Investig..

[28] Coppola D., Nebozhyn M., Khalil F., Dai H., Yeatman T., Loboda A., Mulé J.J. (2011). Unique Ectopic Lymph Node-like Structures Present in Human Primary Colorectal Carcinoma Are Identified by Immune Gene Array Profiling. Am. J. Pathol..

[29] Bindea G., Mlecnik B., Tosolini M., Kirilovsky A., Waldner M., Obenauf A.C., Angell H., Fredriksen T., Lafontaine L., Berger A. (2013). Spatiotemporal Dynamics of Intratumoral Immune Cells Reveal the Immune Landscape in Human Cancer. Immunity.

[30] Rooney M.S., Shukla S.A., Wu C.J., Getz G., Hacohen N. (2015). Molecular and Genetic Properties of Tumors Associated with Local Immune Cytolytic Activity. Cell.

[31] Gatza M.L., Lucas J.E., Barry W.T., Kim J.W., Wang Q., Crawford M.D., Datto M.B., Kelley M., Mathey-Prevot B., Potti A. (2010). A Pathway-Based Classification of Human Breast Cancer. Proc. Natl. Acad. Sci. USA.

[32] Jiang M., Chen J., Zhang W., Zhang R., Ye Y., Liu P., Yu W., Wei F., Ren X., Yu J. (2017). Interleukin-6 Trans-Signaling Pathway Promotes Immunosuppressive Myeloid-Derived Suppressor Cells via Suppression of Suppressor of Cytokine Signaling 3 in Breast Cancer. Front. Immunol..

[33] Sasi W., Jiang W.G., Sharma A., Mokbel K. (2010). Higher Expression Levels of SOCS 1,3,4,7 Are Associated with Earlier Tumour Stage and Better Clinical Outcome in Human Breast Cancer. BMC Cancer.

[34] Sun M., Tang C., Liu J., Jiang W., Yu H., Dong F., Huang C., Rixiati Y. (2021). Comprehensive Analysis of Suppressor of Cytokine Signaling Proteins in Human Breast Cancer. BMC Cancer.

[35] Ghafouri-Fard S., Oskooei V.K., Azari I., Taheri M. (2018). Suppressor of Cytokine Signaling (SOCS) Genes Are Downregulated in Breast Cancer. World J. Surg. Oncol..

[36] Jacquemier J., Bertucci F., Finetti P., Esterni B., Charafe-Jauffret E., Thibult M.-L., Houvenaeghel G., Van den Eynde B., Birnbaum D., Olive D. (2012). High Expression of Indoleamine 2,3-Dioxygenase in the Tumour Is Associated with Medullary Features and Favourable Outcome in Basal-Like Breast Carcinoma. Int. J. Cancer.

[37] Asadzadeh Z., Mohammadi H., Safarzadeh E., Hemmatzadeh M., Mahdian-shakib A., Jadidi-Niaragh F., Azizi G., Baradaran B. (2017). The Paradox of Th17 Cell Functions in Tumor Immunity. Cell. Immunol..

[38] Karpisheh V., Ahmadi M., Abbaszadeh-Goudarzi K., Mohammadpour Saray M., Barshidi A., Mohammadi H., Yousefi M., Jadidi-Niaragh F. (2022). The Role of Th17 Cells in the Pathogenesis and Treatment of Breast Cancer. Cancer Cell Int..

[39] Schmid P., Cortes J., Dent R., Pusztai L., McArthur H., Kümmel S., Bergh J., Denkert C., Park Y.H., Hui R. (2022). Event-Free Survival with Pembrolizumab in Early Triple-Negative Breast Cancer. N. Engl. J. Med..

[40] Delconte R.B., Guittard G., Goh W., Hediyeh-Zadeh S., Hennessy R.J., Rautela J., Davis M.J., Souza-Fonseca-Guimaraes F., Nunès J.A., Huntington N.D. (2020). NK Cell Priming From Endogenous Homeostatic Signals Is Modulated by CIS. Front. Immunol..

[41] Rautela J., Huntington N.D. (2017). IL-15 Signaling in NK Cell Cancer Immunotherapy. Curr. Opin. Immunol..

[42] Pipeline—ONK Therapeutics. https://www.onktherapeutics.com/pipeline/.

