# Supplementary material for: CISH Expression Is Associated with Metastasis-Free Interval in Triple-Negative Breast Cancer and Refines the Prognostic Value of PDL1 Expression
Source: Cancers (Basel). 2022 Jul 10;14(14):3356. doi: 10.3390/cancers14143356 (PMC9316839; doi:10.3390/cancers14143356)
Supplement: Supplementary file 1 [file cancers-14-03356-s001.zip › Supplementary Figure.pdf]

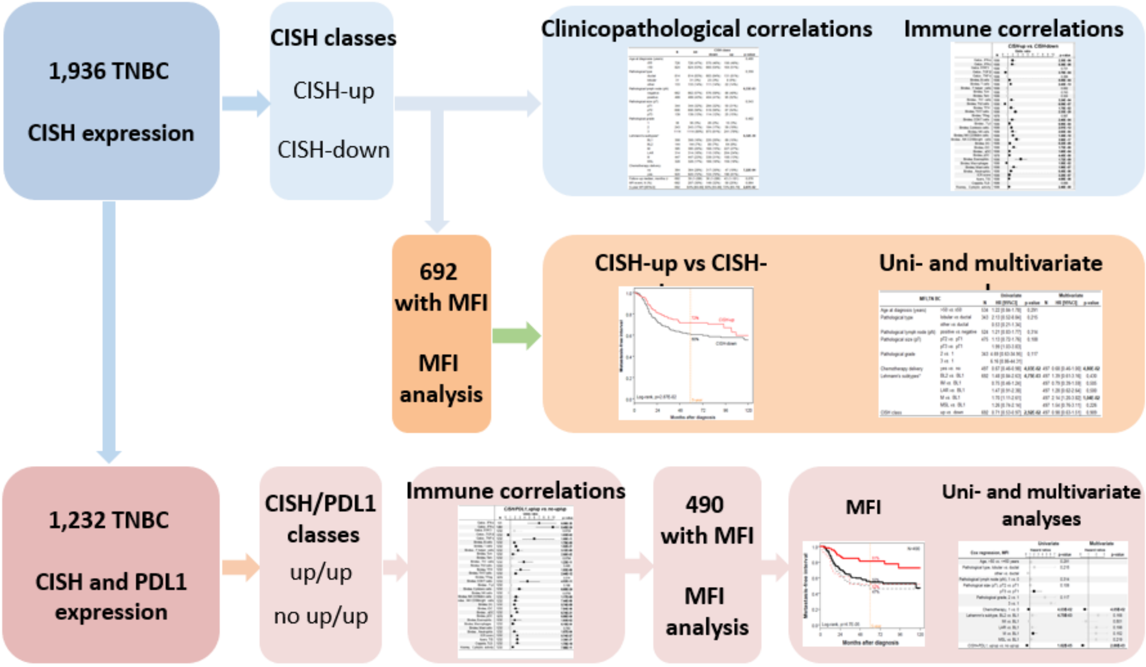

Figure S1. Diagram of analytic workflow.

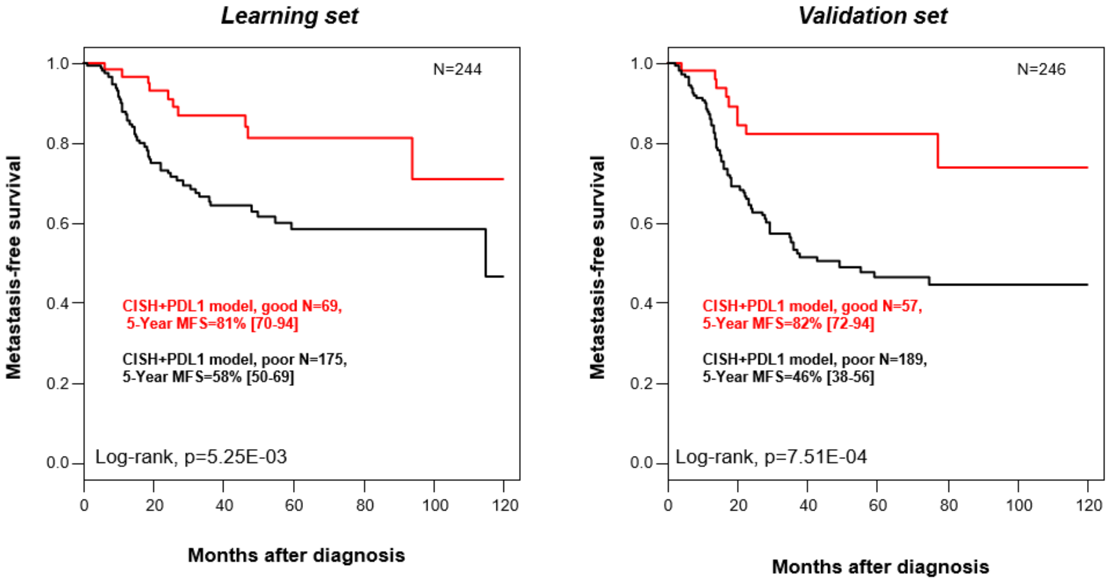

Figure S2. Robustness of the prognostic synergy of CISH and PDL1. The 490 informative samples were divided into two sets, training (244 samples) and validation (246 samples) sets, in which the prognostic value of the CISH/PDL1 expression-based model was tested independently.
